# Management of post-hyperventilation apnea during dental treatment under monitored anesthesia care with propofol

**DOI:** 10.1186/s13030-014-0026-9

**Published:** 2014-12-04

**Authors:** Masato Kobayashi, Shinji Kurata, Takuro Sanuki, Ichiro Okayasu, Takao Ayuse

**Affiliations:** Department of Dental Anesthesiology, Nagasaki University Hospital, 1-7-1 Sakamoto, Nagasaki-shi, 852-8588 Japan; Department of Translational Medical Sciences, Division of Clinical Physiology, Nagasaki University Graduate School of Biomedical Sciences, Course of Medical and Dental Sciences, 1-7-1 Sakamoto, Nagasaki-shi, 852-8588 Japan

**Keywords:** Post-hyperventilation apnea, Propofol, Dental treatment

## Abstract

Although hyperventilation syndrome generally carries a good prognosis, it is associated with the risk of developing severe symptoms, such as post-hyperventilation apnea with hypoxemia and loss of consciousness. We experienced a patient who suffered from post-hyperventilation apnea. A 17-year-old female who suffered from hyperventilation syndrome for several years developed post-hyperventilation apnea after treatment using the paper bag rebreathing method and sedative administration during a dental procedure. We subsequently successfully provided her with monitored anesthesia care with propofol. Monitored anesthesia care with propofol may be effective for the general management of patients who have severe hyperventilation attacks and post-hyperventilation apnea. This case demonstrates that appropriate emergency treatment should be available for patients with hyperventilation attacks who are at risk of developing post-hyperventilation apnea associated with hypoxemia and loss of consciousness.

## Background

Hyperventilation syndrome in patients with no underlying organic abnormality is frequently observed during medical and dental practice. Although the pathophysiological mechanisms of hyperventilation syndrome are still not fully understood, the relative roles of peripheral and central chemoreceptors in causing hyperventilation attacks have been suggested. Although it is believed that episodes of hyperventilation attacks resolve spontaneously and that the paper-bag rebreathing method or administration of anxiolytic agents may help mitigate an attack, sustained symptoms associated with hypocapnia have been reported to initiate complex clinical complications, such as the delayed occurrence of hypoxemia. Furthermore, several case reports have described the occurrence of post-hyperventilation apnea in association with sustained cyanosis, hypoxemia and loss of consciousness [1-4]. The pathogenesis of post-hyperventilation apnea has been linked to the activity of peripheral chemoreceptors, which may contribute to the susceptibility to apnea during hypoxia or hyperoxia [5]. We provided monitored anesthesia care thrice to a patient who had a history of hyperventilation attacks and post-hyperventilation apnea during dental treatment under sedation with regional anesthesia. The aim of this report is to describe the management of hyperventilation attacks and post-hyperventilation apnea during dental treatment.

## Case presentation

### First episode (Figure 1)

**Figure 1:**
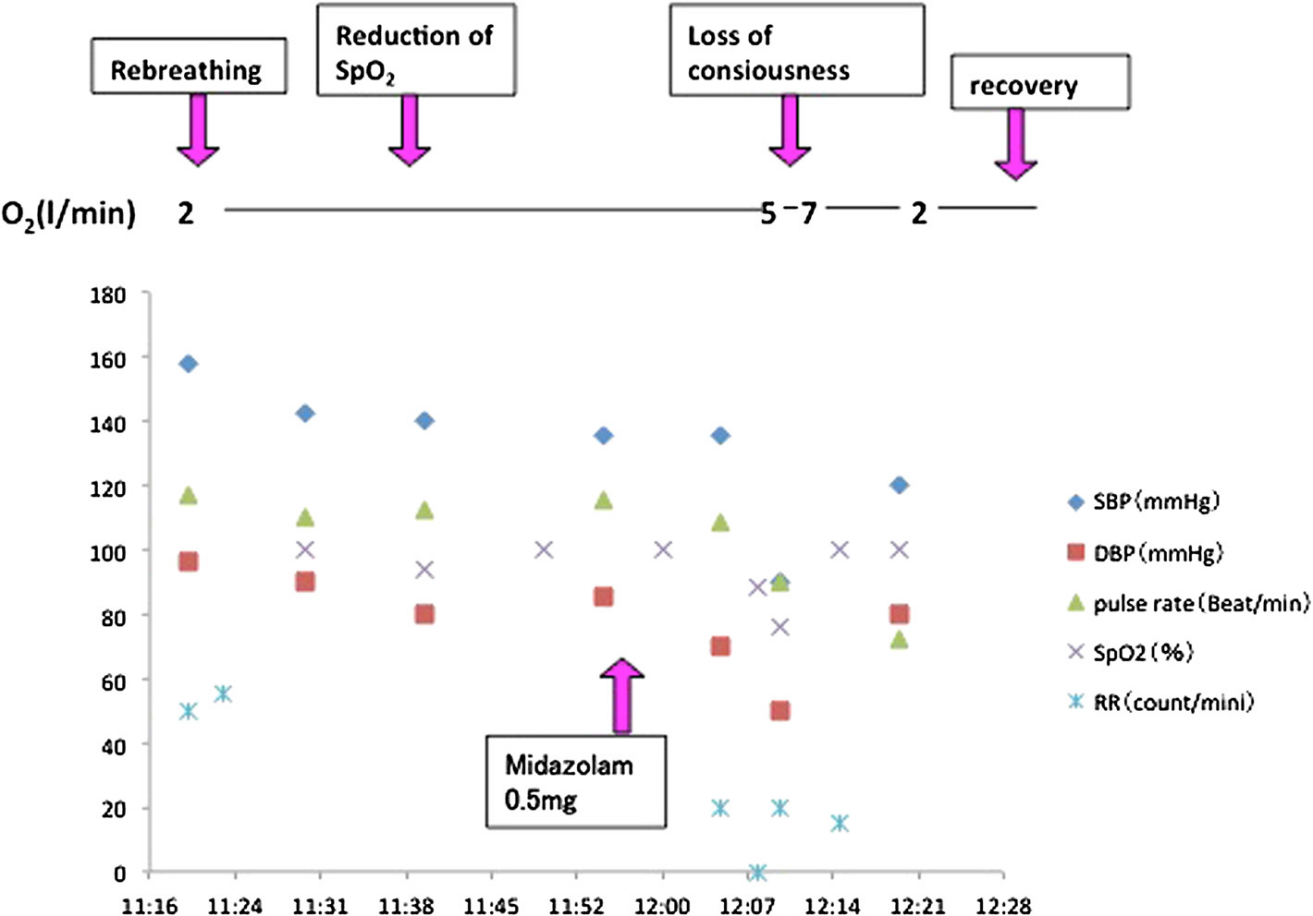
**Time course of the first episode.** Midazolam was used for the treatment of hyperventilation syndrome.

A female 17-year-old (height 154 cm, weight 56.4 kg) was scheduled for dental treatment (root canal treatment for an infected lower second molar) under regional anesthesia at the dental office of a university hospital. Since she had previously experienced hyperventilation attacks, not only during dental treatment but also in other situations, such as during bus rides, she was prescribed benzodiazepines (alprazolam) and paroxetine hydrochloride hydrate by a psychiatrist for a diagnosis of hyperventilation syndrome. There was no other medical history other than the psychological aspects. During the root canal treatment, the patient had a hyperventilation attack after experiencing pain. After fifteen minutes of therapeutic treatment using the rebreathing method with a paper bag did not alleviate her symptoms, our team dental anesthesiologist was asked to support her respiratory condition. As shown in Figure 1, after confirming sustained hyperventilation of respiratory rate 50 ~ 60 breaths/minute with desaturation to 92% associated with blood pressure 130/83 mmHg and pulse rate 115 beats/minute, we decided to provide low dose 2 l/min of oxygen supplementation using a bag-valve-mask instead of using the paper bag. With this, although her oxygen saturation improved to 100%, hyperventilation continued for 20 minutes. Confirming sustained hyperventilation, we decided to administer midazolam intravenously, as generally recommended in the treatment algorithm for hyperventilation syndrome [6]. However, we decided to decrease the dose of intravenous administration of midazolam to 0.5 mg, to minimize the risk of unpredictable changes in respiratory function. Immediately after injection of midazolam the hyperventilation subsided, with her respiratory rate improving to 20 breaths/minute. However, she then lost consciousness and developed complete apnea, resulting in significant desaturation to 88 ~ 76%. We tried to treat her with artificial ventilation using a bag-valve-mask with supplementation of a higher dose of 5 ~ 7 l/min oxygen. Two minutes after continuous mask ventilation of 8 ~ 10 counts/min to treat the complete apnea associated with unconsciousness and cyanosis, she regained consciousness and began to breathe at a respiratory rate of 15 breaths/minute. Her blood pressure was 120/80 mmHg and pulse rate was 72 beats/minute. We continuously monitored her respiratory condition for ne hour until full recovery from the symptoms. Thereafter, we informed the patient and her mother about the episode of severe hyperventilation attack and post-hyperventilation apnea during the dental treatment and recommended that she undergo further such treatments under monitored anesthesia, care with spontaneous breathing.

### Second episode (Figure 2)

**Figure 2:**
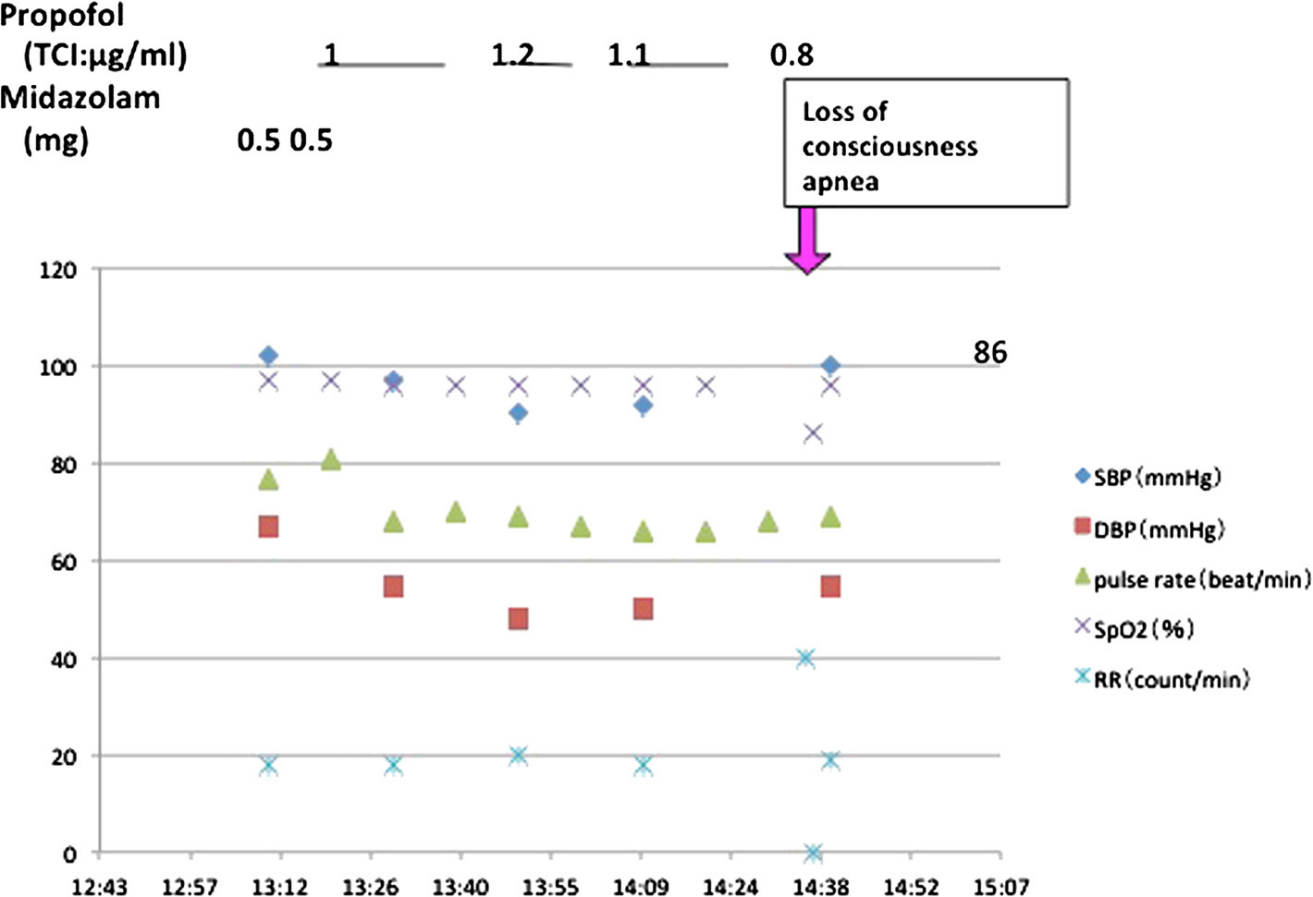
**Time course of the second episode.** Propofol anesthesia after midazolam premedication was used for general management.

Several weeks later, the same patient was scheduled for dental treatment under monitored anesthesia care with spontaneous breathing plus regional anesthesia. We induced and maintained anesthesia for one hour with a target controlled infusion of propofol (TCI level = 1.1 ~ 1.2 μg/ml) after intravenous administration of 1 mg midazolam. At the end of treatment, propofol was discontinued and the patient was carefully observed by the anesthesiologist until return of consciousness. Although she regained consciousness 20 minutes after stopping the propofol infusion, hyperventilation with a respiratory rate of 40 breaths/minute occurred and continued for several minutes. Immediately after the hyperventilation subsided spontaneously, post-hyperventilation apnea with desaturation to 86% occurred in association with loss of consciousness. However, the patient regained consciousness within one minute without any therapeutic intervention. She was discharged after confirming full recovery from the unstable respiratory condition.

### Third episode (Figure 3)

**Figure 3:**
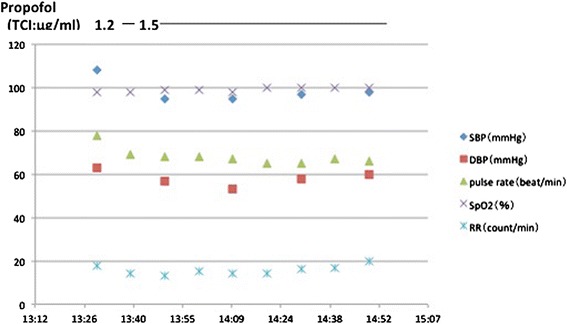
**Time course of the third episode.** Propofol was used for general management.

The patient was scheduled for the third dental treatment session under monitored anesthesia care with spontaneous breathing plus regional anesthesia. As with the previous anesthetic management, we induced without any premedication and maintained anesthesia with a TCI of propofol (TCI level = 1.2 ~ 1.5 μg/ml) for the fifty minute treatment period. At the end of the treatment, propofol was discontinued and the anesthesiologist carefully observed the patient until recovery of consciousness. She did not develop any symptoms of hyperventilation, even 10 minutes after regaining consciousness and was later discharged after an uneventful recovery period.

## Discussion

It is known that some patients with hyperventilation syndrome develop post-hyperventilation apnea or hypoxia with cyanosis and loss of consciousness, resulting in severe complications, including death [7,8]. The pathogenesis of post-hyperventilation apnea has only been partly understood as being due to the activity of peripheral chemoreceptors that may contribute to the susceptibility to apnea during both hypoxia and hyperoxia [2,5,9]. Ogawa et al. suggested that apnea secondary to hyperventilation occurs because following the wash out of carbon dioxide from the body during hyperventilation an extended period of time is required for it to accumulate adequately enough to reach the threshold required to stimulate breathing [10]. In our patient, because oxygen continued to be supplied during artificial respiration with bag-valve-mask, even after the hyperventilation diminished, the patient probably had a low PaCO_2_ and high PaO_2_. In such a situation, the imbalance in the levels of both PaO_2_ and PaCO_2_ would have resulted in the peripheral chemoreceptors initiating a compensatory apnea reaction until sufficient carbon dioxide accumulated to reach the threshold to stimulate adequate breathing. Although, usually, a vicarious arousal reaction to the apnea can occur, in this case this arousal reaction was probably inhibited by the sedative effect of the small dose of midazolam used in first episode and second episodes.

It has also been suggested that administration of benzodiazepine drugs, such as diazepam or midazolam, may contribute to increasing the threshold for respiratory stimulation. In other words, reduction of respiratory control related to the sedative effects of drugs might decrease the respiratory stimulant effect of arterial carbon dioxide wash out [11]. They suggest that 5-hydroxytryptamine 1A agonists can provide adequate anxiolysis in patients who have frequent occurrence of post-hyperventilation apnea. These studies indicate that the drugs used to manage and treat patients with hyperventilation syndrome should be carefully chosen, due to the risk of occurrence of post-hyperventilation apnea. In our case, we used midazolam followed by propofol, a short acting intravenous anesthetic agent, during the second episode. However, when we did not use midazolam in the third episode, there were no symptoms of post-hyperventilation apnea after propofol anesthesia. We cannot deny that the small dose of midazolam administered may have caused the post-hyperventilation apnea due to its sedative effect. However, it is difficult to believe that the effects of 1 mg administration of midazolam would remain active for the 1 hour 20 minutes before the emergence of post-hyperventilation apnea. The exact influence of propofol on the occurrence of post-hyperventilation apnea is not known. Previously, Tomioka et al. reported that propofol was not effective in preventing hyperventilation syndrome [12]. However, based on the third episode in our patient, in which she regained consciousness from propofol anesthesia without any symptoms of hyperventilation and/or post-hyperventilation apnea, we believe that an adequate dose of propofol is useful in patients who frequently experience hyperventilation syndrome.

The timing of drug usage for sustained hyperventilation in the case of drugs that influence the occurrence of apnea also needs discussion. The duration for which carbon dioxide rebreathing using a paper bag should be attempted before further intervention should be determined. Treatment using a rebreathing bag for a prolonged period may result in a decrease in oxygen content. In our case, a cause of the desaturation (92%) might be related to reducing the oxygen supply by inappropriate continuation of rebreathing paper bag for a prolonged period of 15 minutes before the arrival of our team. Callaham et al. revealed that the paper bag rebreathing method seems to be a potentially risky procedure, because death, severe hypoxia and collapse have been reported to occur with paper bag rebreathing [7]. The need for sedative/anxiolytic drug therapy in the early stages of hyperventilation should be considered, even though sedative and anxiolytic agents have the potential to depress respiratory control. Another unanswered question is the need for oxygen supplementation if desaturation is observed during rebreathing therapy, and whether the arterial PO_2_ level directly alters the compensatory response to hyperventilation. Previous reports have suggested that it might be desirable to provide a proper amount of oxygen, because hypoxemia is detrimental for the brain and hypoxemia during hypocapnia does not stimulate respiration [1]. Our experience seems to indicate that respiratory assistance using a bag and mask should be performed in critical cases of post-hyperventilation apnea with hypoxemia and loss of consciousness. It should be noted that most patients with post-hyperventilation apnea recover spontaneously in a short time without any treatment. Therefore, we can wait for spontaneous recovery for some time without the use of a bag-valve-mask under full monitoring of vital signs related to cardio-respiratory function. However, as indicated in a previous case report by Munemoto T et al. [1], if prolonged post-hyperventilation apnea associated with severe hypoxemia persists, as a precautionary measure it is necessary to immediately perform respiratory assistance by bag-valve-mask. Routine monitoring of SpO_2_ and other vital signs, such as heart rate and blood pressure, should be available for patients with hyperventilation syndrome.

## Conclusion

We experienced a case of post-hyperventilation apnea during dental treatment. Our experience suggests that monitored anesthesia care with propofol may be effective in the general management of patients with severe hyperventilation attacks and post-hyperventilation apnea. This case highlights the fact that proper emergency treatment should be available for patients with hyperventilation syndrome who might develop post-hyperventilation apnea associated with hypoxemia and loss of consciousness.

## Consent

Written informed consent was obtained from the patient’s parent for the publication of this case report and accompanying images.
